# Combining GAL4 GFP enhancer trap with split luciferase to measure spatiotemporal promoter activity in Arabidopsis

**DOI:** 10.1111/tpj.14603

**Published:** 2019-12-03

**Authors:** Ángela Román, John F. Golz, Alex A. R. Webb, Ian A. Graham, Michael J. Haydon

**Affiliations:** ^1^ School of BioSciences University of Melbourne Melbourne Australia; ^2^ Department of Biology University of York York United Kingdom; ^3^ Department of Plant Sciences University of Cambridge Cambridge United Kingdom

**Keywords:** gene expression, luciferase, enhancer trap, circadian clock, tissue‐specificity, Arabidopsis, technical advance

## Abstract

In multicellular organisms different types of tissues have distinct gene expression profiles associated with specific function or structure of the cell. Quantification of gene expression in whole organs or whole organisms can give misleading information about levels or dynamics of expression in specific cell types. Tissue‐ or cell‐specific analysis of gene expression has potential to enhance our understanding of gene regulation and interactions of cell signalling networks. The Arabidopsis circadian oscillator is a gene network which orchestrates rhythmic expression across the day/night cycle. There is heterogeneity between cell and tissue types of the composition and behaviour of the oscillator. In order to better understand the spatial and temporal patterns of gene expression, flexible tools are required. By combining a Gateway®‐compatible split luciferase construct with a *GAL4 GFP* enhancer trap system, we describe a tissue‐specific split luciferase assay for non‐invasive detection of spatiotemporal gene expression in Arabidopsis. We demonstrate the utility of this enhancer trap‐compatible split luciferase assay (ETSLA) system to investigate tissue‐specific dynamics of circadian gene expression. We confirm spatial heterogeneity of circadian gene expression in Arabidopsis leaves and describe the resources available to investigate any gene of interest.

## Significance Statement

Cell and tissue type resolution of gene expression is important to understand functional properties of signalling networks. We have adapted a split luciferase system for use with existing collections of enhancer trap lines to provide a versatile tool to detect dynamic tissue‐specific gene expression in whole Arabidopsis seedlings and demonstrate its utility to detect spatial heterogeneity of circadian rhythms in leaves.

## Introduction

In multicellular organisms different cell and tissue types have distinct gene expression profiles. Quantification of gene expression in whole organs or whole organisms can give misleading information about levels or dynamics of expression in specific cell types. Tissue‐ or cell‐specific analyses of gene expression can enhance our understanding of transcriptional responses to environmental cues. The circadian clock orchestrates rhythmic gene expression according to daily environmental cues. The Arabidopsis core circadian oscillator is comprised of a gene network of regulatory feedback loops involving around 20 genes (Haydon *et al.*, 2019). Although most core oscillator genes are expressed in all cells, there is spatial heterogeneity of circadian gene expression in Arabidopsis (Para *et al.*, 2007; Xu *et al.*, 2007; James *et al.*, 2008; Wenden *et al.*, 2012; Martí *et al.*, 2013; Endo *et al.*, 2014; Takahashi *et al.*, 2015; Bordage *et al.*, 2016; Kim *et al.*, 2016; Gould *et al.*, 2018; Greenwood *et al.*, 2019).

Several techniques have been used to isolate specific cell populations from plant tissues for gene expression analyses but a common disadvantage of these is the destructive nature of sampling. Sampling protocols can alter gene expression, diminish intercellular signalling, and limit resolution for temporal information. Laser‐Capture Microdissection (LCM) was developed to isolate cell populations from sections of animal tissues for gene expression analyses (Emmert‐Buck *et al.*, 1996; Bonner *et al.*, 1997) and has been similarly applied to plant tissues (Asano *et al.*, 2002; Kerk *et al.*, 2003; Nakazono *et al.*, 2003). RNA can be extracted from LCM samples and used for quantitative transcript analyses. Using LCM in Arabidopsis, circadian rhythms have been measured in shoot apices by measuring transcripts and fluorescent proteins in dissected tissues (Takahashi *et al.*, 2015). However, this technique is limited by accessibility of the tissue of interest and identifiable cell types. Similarly, a protocol was developed to isolate mesophyll, vasculature, and epidermal tissues from Arabidopsis leaves with high purity to measure transcripts over a circadian time course, which indicated distinct characteristics of circadian gene expression in leaf vasculature (Endo *et al.*, 2014).

Transcriptome analyses of fluorescence activated cell sorting (FACS) of protoplasted transgenic plants expressing cell type‐specific GFP markers has allowed high‐resolution spatial maps of transcription in Arabidopsis roots (Birnbaum *et al.*, 2003; Brady *et al.*, 2007; Dinneny *et al.*, 2008), and more recently adapted for leaves (Grønlund *et al.*, 2012; Coker *et al.*, 2015). However, the process of protoplasting can alter gene expression. By contrast, the INTACT method (isolation of nuclei tagged in specific cell types) uses an affinity approach to isolate nuclei from transgenic plants expressing a cell type‐specific biotinylated nuclear protein marker (Deal and Henikoff, 2011). This method has been used to determine cell type‐specific nuclear transcriptomes in numerous plant species to provide spatial information about gene expression in diverse cell types (Ron *et al.*, 2014; Moreno‐Romero *et al.*, 2017; Del Toro‐De Leon and Kohler, 2018; Reynoso *et al.*, 2018). However, INTACT has so far not been applied to measure temporal characteristics of gene expression.

Emerging single‐cell RNA sequencing (scRNA‐seq) technologies have the potential to generate high‐resolution maps of gene expression networks, particularly in emerging model species where specific fluorescent markers are not available (Efroni and Birnbaum, 2016). Recent studies have performed scRNA‐seq on protoplasts from Arabidopsis root cells using droplet‐based microfluidics to provide the first gene expression maps of roots of wild‐type and seedlings at single‐cell resolution (Ryu *et al.*, 2019; Denyer *et al.*, 2019; Jean‐Baptiste *et al.*, 2019). These studies have provided high‐resolution spatiotemporal maps and identified developmental waves of gene expression associated with root cell differentiation. Applications of scRNA‐seq in plant systems are likely to accelerate as the technology and data analyses become more accessible.

Transgenic luciferase reporters are well suited for measuring gene expression *in planta* with high temporal resolution. However, luminescence imaging systems typically have poor spatial resolution. A modified split luciferase system has been shown to be effective for measuring circadian rhythms specifically in phloem companion cells (Endo *et al.*, 2014). The N‐ and C‐terminal halves of luciferase were expressed from a phloem‐specific and circadian gene promoter, respectively, and the reconstituted luciferase produces luminescence only in the phloem companion cells in which both transgenes are expressed. This system allows measurement of gene expression in specific cell and tissue types but depends on availability of characterized tissue‐specific promoters.

Enhancer trap screens have been effective in identifying tissue‐specific enhancer elements in Arabidopsis. Rather than reporting activity of a full promoter, which can be regulated by multiple endogenous and environmental signals, enhancer trap lines drive reporter activity from a specific enhancer. In this way, these lines can be advantageous over tissue‐specific promoters. The *GAL4 GFP* enhancer trap lines carry a transgene encoding a GAL4‐VP16 transcriptional activator from yeast with a minimal promoter and a modified GFP targeted to the ER (mGFP5ER) under the control of GAL4‐binding upstream activation sequences (*UAS*). Transformants have been screened for diverse spatial patterns of GFP fluorescence, driven by enhancer elements in the vicinity of the insertion regulating *GAL4‐VP16* (Haseloff, 1999; Laplaze *et al.*, 2005). Unique patterns of expression have been identified for enhancer trap lines for which there is no known gene promoter (Gardner *et al.*, 2009). Characterized enhancer trap lines can be used to drive tissue‐specific expression of any gene of interest from a *UAS* by introducing a second transgene by crossing or transformation. In this way, *GAL4 GFP* enhancer trap lines have been exploited to modify gene expression in specific cell types (Laplaze *et al.*, 2005; Laplaze *et al.*, 2007; Gan *et al.*, 2012) or drive cell type‐specific reporters (Dodd *et al.*, 2006; Jia *et al.*, 2007; Martí *et al.*, 2013) by transactivation. There are *c.* 250 *GAL4 GFP* enhancer trap lines across four sets available from stock centres and characterization of these lines continues to increase, broadening their utility (Ckurshumova *et al.*, 2009; Radoeva *et al.*, 2016).

We have exploited the tissue‐specific variation of the circadian oscillator to develop and test the combination of a Gateway®‐compatible split luciferase construct with an established GAL4 enhancer trap system (Laplaze *et al.*, 2005) for non‐invasive detection of spatiotemporal gene expression in Arabidopsis. We demonstrate the utility of this enhancer trap split luciferase assay (ETSLA) system to investigate tissue‐specific promoter activity and apply this to measure circadian gene expression. We confirm spatial heterogeneity of circadian promoter activity in Arabidopsis leaves and describe the resources available to investigate any gene of interest.

## Results

### Development of an ETSLA system

**Figure 1:**
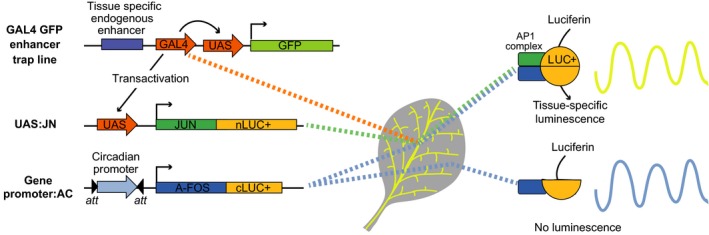
Components of the enhancer trap tissue‐specific split luciferase assay system. In the *GAL4 GFP* enhancer trap lines, expression of *GAL4* is driven by a minimal promoter in the transgene and a tissue‐specific enhancer in the genome. GAL4, a yeast transcriptional activator, binds to upstream activation sequences (*UAS*) to produce tissue‐specific expression of GFP, which is encoded by the same transgene. The split luciferase system requires the introduction of two transgenes. In one, *UAS* is upstream of a sequence coding a fusion product of an N‐terminal region of luciferase and a c‐Jun subunit of the AP1 complex. In the second, a promoter of interest is inserted between *att* sites by Gateway® cloning upstream of a sequence for a fusion of the C‐terminal region of luciferase and an A‐Fos subunit of the AP1 complex. Co‐expression of all three transgenes in a cell (for example, in leaf vasculature) allows reconstitution of a functional luciferase enzyme, facilitated by the stable interaction of the AP1 complex subunits, generating tissue‐specific luminescence (yellow line). Cells in which the tissue‐specific enhancer is not activated express only the C‐terminal half of luciferase (blue line) and do not produce luminescence.

To measure gene expression in specific tissues in Arabidopsis we set out to adapt a split luciferase system to be applicable with existing *GAL4 GFP* enhancer trap lines (Figure 1). Enhancer trap lines are available which contain a transgene including a minimal promoter driving *GAL4* expression inserted adjacent to endogenous or cryptic tissue‐specific enhancer elements in the Arabidopsis genome. The transgene also includes *UAS* elements driving *GFP* to localize expression of *GAL4* and verify transactivation of *UAS*. In order to exploit these *GAL4 GFP* enhancer trap lines for tissue‐specific luciferase, we generated two constructs. A UAS:JN construct comprising a *UAS* upstream of a fusion of an N‐terminal half of LUCIFERASE+ (nLUC+) and the c‐Jun bZIP domain of a heterodimer of the AP1 complex. A GW:AC construct comprises a Gateway® cassette upstream of a fusion of a C‐terminal half of LUC+ and A‐Fos, a leucine zipper domain which interacts with c‐Jun (Endo *et al.*, 2014). In principle, when both halves of LUC+ are expressed in the same cell, the stable formation of the AP1 complex will reconstitute the luciferase enzyme and emit bioluminescence in the presence of its substrate, D(+)‐luciferin.

**Figure 2:**
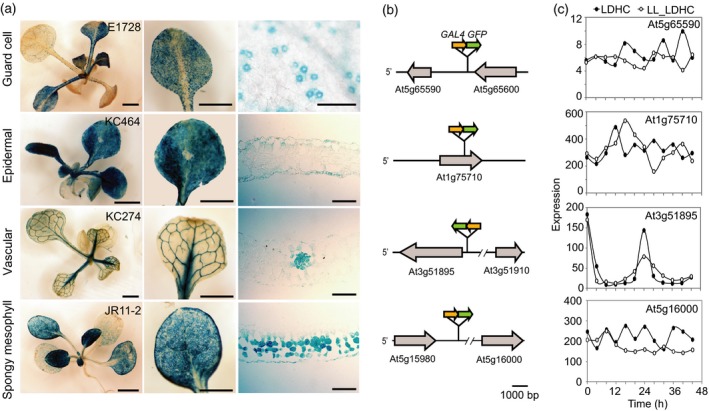
Characterization of *GAL4 GFP* enhancer trap lines used in this study. (a) GUS staining of 20‐day‐old T1 transformants of *UAS:GUS* in *GAL4 GFP* enhancer trap lines in guard cells (E1728), leaf epidermis (KC464), vasculature (KC274) and spongy mesophyll (JR11‐2). Leaf cross‐sections are shown for the epidermal, vascular and mesophyll lines (right). Bars represent 1 mm (left, middle) or 100 µm (right). (b) Genomic location and orientation of the *GAL4 GFP* T‐DNAs. Flanking protein coding genes are shown. Arrows indicate direction of transcription. (c) Expression of transcripts adjacent to each *GAL4 GFP* T‐DNA in diel (LDHC) or continuous light (LL_LDHC) conditions. Data were obtained from diurnal.mocklerlab.org (Mockler *et al*., 2007).

The *GAL4 GFP* enhancer trap system has been adapted to Arabidopsis (Haseloff, 1999) and numerous lines have been reported and characterized (Laplaze *et al.*, 2005; Radoeva *et al.*, 2016) and are available from seed stock centres (Table S1). As a proof of concept, we chose four Arabidopsis enhancer trap lines with distinct patterns of expression in the leaf. These lines have tissue‐specific GFP expression in spongy mesophyll (JR11‐2), leaf vasculature (KC274), leaf epidermis (KC464) and guard cells (E1728), as previously shown with confocal laser scanning microscopy (Gardner *et al.*, 2009; Martí *et al.*, 2013). To confirm that these lines can drive transactivated expression of a reporter in a second transgene in the expected tissues, we introduced a *UAS:β‐GLUCURONIDASE *(*GUS*) construct by transformation. For all four *GAL4 GFP* lines, strong GUS activity was consistently detected in the expected tissue or cell types, (Figure 2a). Weak GUS staining was sometimes detected in neighbouring cells, which we interpret as diffusion of the reaction product. The expected GUS patterns were observed in 50/52 T1 seedlings, representing the four lines. This suggests tissue‐specific transactivation is robust in these *GAL4 GFP* enhancer trap lines.

In order to further characterize these lines, we used Thermal Asymmetric InterLaced (TAIL) PCR (Liu and Whittier, 1995) to identify the positions of the T‐DNA inserts in the genome (Figure 2b). We confirmed the T‐DNA in E1728 (guard cell *GAL4 GFP* enhancer trap line) on chromosome 5 (position 26215578) flanked by the coding sequences of a putative chloroplast‐targeted Dof zinc finger transcription factor (At5g65590; *STOMATAL CARPENTER 1, SCAP1*) and a L‐type lectin receptor kinase (At5g65600, *LECRK‐IX.2*), as previously reported (Gardner *et al.*, 2009). The orientation of the *GAL4 GFP* transgene is in the opposite orientation to both flanking genes (Figure 2b). The T‐DNA in KC464 (epidermal *GAL4 GFP* enhancer trap line) was located on chromosome 1 (position 28430296) within the second intron of the gene sequence of a C2H2‐like zinc finger protein (At1g75710), 1490 bp downstream of the start codon in the same orientation as the gene. Thus, *GAL4* might be expressed similarly to the protein coding gene (Figure 2b). The T‐DNA in KC274 (vascular *GAL4 GFP* enhancer trap line) is located on chromosome 3 (position 19256215), flanked by the coding sequences of a chloroplast‐localized sulphate transporter (At3g51895; *SULTR3;1*) and a heat shock transcription factor (At3g51910; *HSFA7A*). The *GAL4 GFP* transgene is 538 bp upstream of At3g51895 and oriented in the same direction (Figure 2b). The T‐DNA in JR11‐2 (spongy mesophyll *GAL4 GFP* enhancer trap line) is on chromosome 5 (position 5217128), flanked by the coding sequences of a putative member of the pentatricopeptide repeat superfamily (At5g15980) and an NSP‐interacting receptor‐like kinase (At5g16000; *NIK1*). The *GAL4 GFP* transgene is in the same orientation as both flanking genes, upstream of At5g16000 (Figure 2b).

To validate the use of the selected enhancer trap lines for investigation of circadian rhythms of gene expression, we examined the rhythmic expression of the gene adjacent to each enhancer trap locus using Diurnal, a database of published microarray data sets (Mockler *et al.*, 2007; Figure 2c). The expression of At5g65590 (E1728, guard cell), At1g75710 (KC464, epidermal) or At5g16000 (JR11‐2, spongy mesophyll) were not overtly rhythmic in diel (LDHC) or continuous light (LL_LDHC) conditions. Expression of At3g51895 (KC274, vascular) was rhythmic in both diel and continuous light, peaking at dawn.

**Figure 3:**
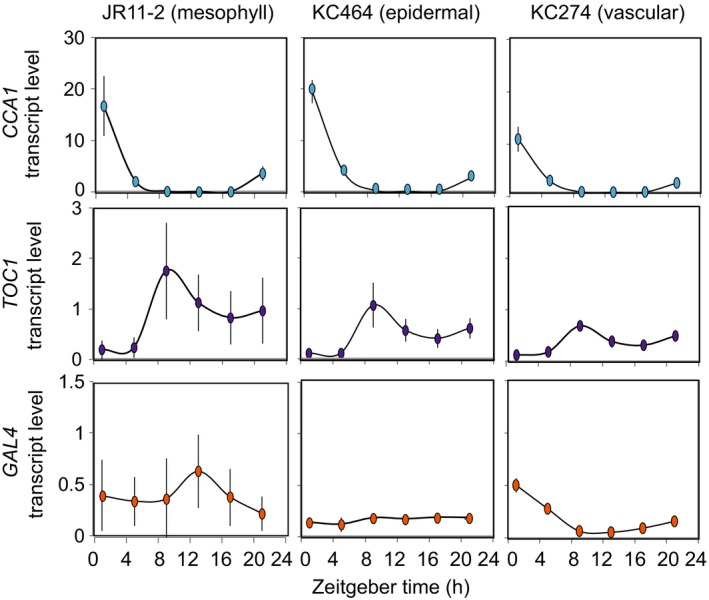
Transcript levels in *GAL4 GFP* enhancer trap lines in diel conditions. Transcript levels of *TOC1*, *CCA1*, and *GAL4*, relative to *IPP2* and *PP2AA3*, in 10–14‐day‐old enhancer trap line seedlings growing under light–dark cycles (*zeitgeber*). Values are means ± SD, *n* = 3.

Expression of the gene adjacent to the *GAL4 GFP* transgene might not be an indicative marker for the activity of the enhancer. Therefore, we directly measured expression of the *GAL4* transcript by quantitative reverse transcriptase polymerase chain reaction (qRT‐PCR) in the epidermal, vascular and spongy mesophyll lines over a 24 h diel cycle (Figure 3). Consistent with the Diurnal data (Figure 2c), diel rhythms of *GAL4* transcript level were not detected in the spongy mesophyll or epidermal lines. Transcript levels of *GAL4* peaked at zeitgeber time (ZT) 0 in the vascular line, although the amplitude was substantially lower than circadian clock genes *CIRCADIAN CLOCK ASSOCIATED 1 (CCA1)* and *TIMING OF CAB2 1 (TOC1)* (Figure 3). The diel oscillation of *GAL4* in this enhancer trap line might alter the rhythms of the reconstituted luciferase in these tissues. Conversely, the relatively low amplitude rhythm of the enhancer might not be sufficient to substantively impact regulation of the UAS in the context of highly expressed circadian clock reporters. In either case, examination of rhythmic expression of *GAL4* in each enhancer line is necessary to interpret estimates of circadian rhythms with this split luciferase system.

The ETSLA system requires the introduction of two transgenes into the *GAL4 GFP* enhancer trap lines. We reasoned that best strategy was to co‐transform the UAS:JN and promoter:AC construct into wild‐type *A. thaliana* by floral dip and then cross the double transformants with each enhancer trap line and measure luminescence in F1 and subsequent generations. The advantage of this strategy is that differences between luciferase signal can be confidently assigned to the enhancer trap by controlling for position effects of the new transgenes. We also used an alternative strategy of transforming the UAS:JN construct into each enhancer trap line and then crossing these double transgenics to each promoter:AC transgenic. The latter strategy has the advantage of versatility of the system for new promoters of interest.

**Figure 4:**
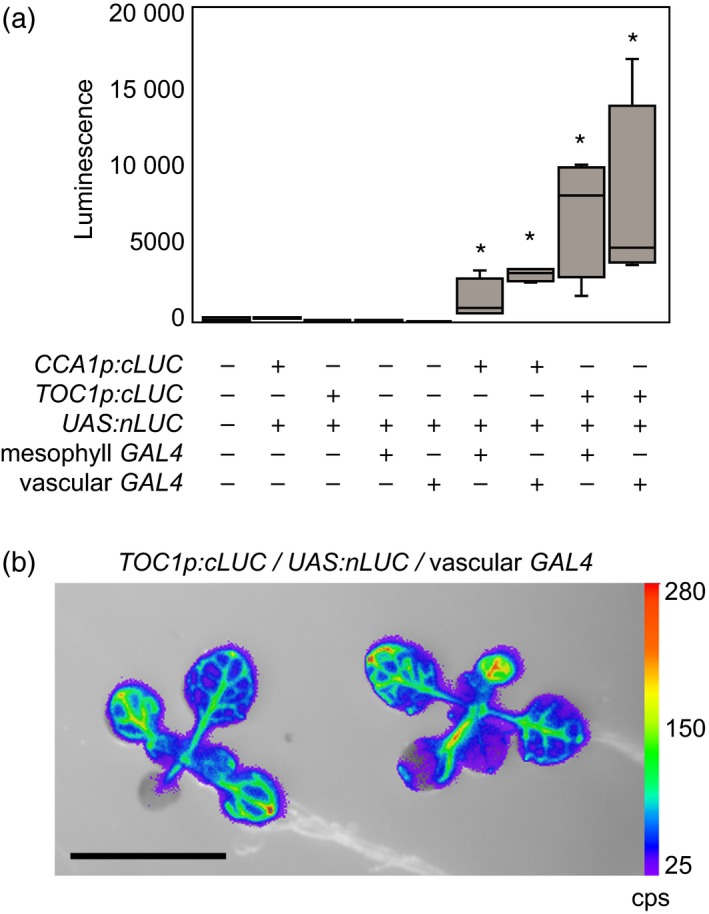
Tissue‐specific luminescence using enhancer trap split luciferase assay (ETSLA). (a) Luciferase luminescence in transgenic seedlings containing combinations of a *CCA1p:AC *(*cLUC*)*, TOC1p:AC* and *UAS:JN *(*nLUC*) transgenes transformed into wild‐type (Col‐0) or *GAL4 GFP* enhancer trap lines. Data are represented as Min–Max box plots, *n* = 4. Asterisks indicate statistical difference from non‐transformed wild‐type by two‐tailed *t‐*test with Bonferroni corrections (*P* < 0.01). (b) Luminescence image overlaid on a bright‐field image of 15‐day‐old seedlings containing *TOC1p:AC* and *UAS:JN* transgenes in the vascular *GAL4 GFP* enhancer trap line (KC274). Bar represents 10 mm. False colour scale represents counts per second (cps).

To test the application of the ETSLA system to measure circadian rhythms in Arabidopsis seedlings, we used the GW:AC vector to generate constructs for three core circadian oscillator genes with distinct phases in the morning (*CCA1),* afternoon *(PSEUDO RESPONSE REGULATOR 7; PRR7)* and evening (*TOC1)* and an evening‐phased circadian output gene (*COLD, CIRCADIAN RHYTHM AND RNA BINDING 2; CCR2*). The promoter:AC and UAS:JN constructs were introduced into the enhancer trap lines by one or both of the alternative strategies described above (Table S2). We identified populations harbouring all three transgenes for the vascular and mesophyll enhancer trap lines and detected luminescence in all lines. We first confirmed that the presence of all three transgenes is both necessary and sufficient to produce luciferase luminescence. We did not detect luminescence signal above background levels in Arabidopsis seedlings containing any two of a *promoter:AC*, *UAS:JN* or a *GAL4 GFP* transgene. Clear signal was only detected when all three transgenes were present (Figure 4a). Luminescence imaging of the *TOC1* vascular ETSLA lines, carrying all three transgenes, indicated a clear vascular pattern of luminescence signal in leaves (Figure 4b). Together, these confirm that the split luciferase enzyme can be effectively reconstituted using the *GAL4 GFP* enhancer trap lines to produce tissue‐specific luminescence in Arabidopsis seedlings.

### Tissue‐specific features of circadian oscillator gene expression

**Figure 5:**
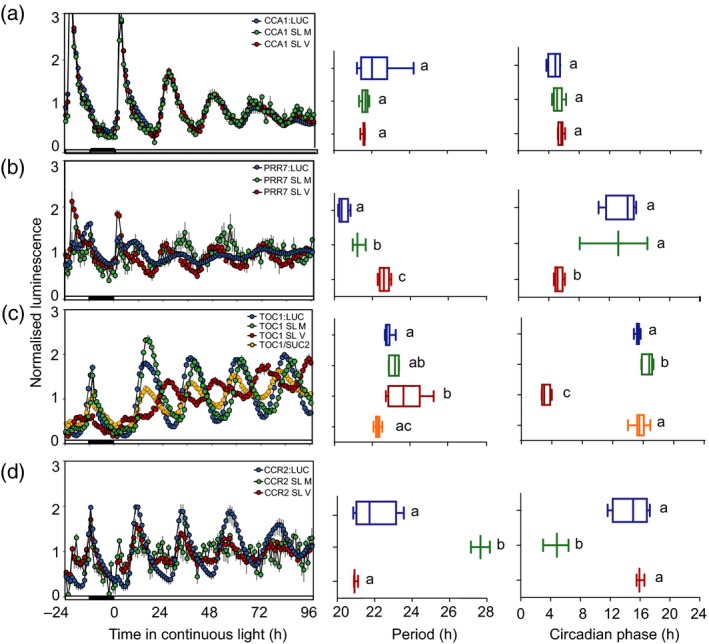
Tissue‐specific circadian clock promoter activity using enhancer trap split luciferase assay (ETSLA). Normalized luminescence, period and phase estimates of luciferase activity in 10–14‐day‐old seedlings in continuous light for (a) *CCA1*, (b) *PRR7,* (c) *TOC1*, and (d) *CCR2* promoters. ETSLA lines for vascular and mesophyll expression are shown, compared with the promoter:LUC control for each gene and the *SUC2*‐driven TSLA line for TOC1. Luminescence values are means ± SEM, *n* = 4–8. Period and phase estimates are represented as Min–Max box plots. Statistical differences from the promoter:LUC control were determined by one‐way anova with Tukey's post‐hoc tests. Different letters indicate significant differences between samples (*P* < 0.05).

To measure circadian rhythms in the ETSLA lines we imaged luminescence in multiple populations (Table S2) harbouring all three transgenes in continuous light and compared them to *promoter:LUC* lines reporting whole‐seedling promoter activity (Figure 5). Robust circadian rhythms were detected for all promoters with a period ranging from 21 to 28 h (mean period 22.3 ± 1.3 h; mean relative amplitude error 0.28 ± 0.15; Table S2). For the *CCA1* promoter, we could not detect a difference in circadian period or phase between the vascular or mesophyll ETSLA lines and *CCA1p:LUC* control (Figure 5a). This suggests circadian rhythms of *CCA1* expression are similar in vascular and mesophyll tissues, as previously reported (Endo *et al.*, 2014).

PRR7p*:*LUC activity comprises two peaks in diel cycles (Figure 5b); a light‐activated peak at dawn followed by a circadian peak in the afternoon. Rhythms of *PRR7* promoter were distinct in mesophyll and vascular tissues. Circadian period was significantly longer in both tissue types compared with whole‐seedling PRR7p:LUC activity, particularly in the vascular ETSLA lines. The circadian phase of the *PRR7* promoter was significantly advanced by about 9 h in the vascular line compared with *PRR7p:LUC*, suggesting distinct oscillator behaviour between these cell types. Interestingly, the earlier peak of *PRR7* in the vascular line coincides with the phase of the light‐activated peak in *PRR7p:LUC*, so might be due to increased light sensitivity of these cells.

The period of *TOC1* promoter activity was similar in the mesophyll ETSLA lines compared with total *TOC1p:LUC* with a small, but significant, phase delay (1.3 h; Figure 5c). By contrast, the period of *TOC1* promoter activity was significantly longer in vascular lines compared with *TOC1p:LUC* and the phase was dramatically advanced, similar to *PRR7*. Differences in circadian rhythms of *TOC1* promoter have previously been reported when driven ubiquitously or in phloem companion cells from the *SUCROSE‐PROTON SYMPORTER 2 (SUC2)* promoter (Endo *et al.*, 2014), so we directly compared rhythms in *SUC2p/TOC1p* TSLA with the vascular ETSLA lines (Figure 5c). We did not detect a significant difference in circadian period or phase between the SUC2p/TOC1p TSLA lines compared with *TOC1p:LUC* or the *TOC1* mesophyll ETSLA lines in our experiments*.* The differences in luciferase rhythms between the SUC2p/TOC1p TSLA and the TOC1 vascular ETSLA lines might be due to differences in tissue‐specificity of these two lines. The *SUC2* promoter is specifically active in phloem companion cells (Truernit and Sauer, 1995; Schulze *et al.*, 2003), whereas we detected broad expression throughout leaf vascular bundles from the vascular enhancer (Figure 2a).

We considered whether the earlier phase in the vascular ETSLA lines for both *TOC1* and *PRR7* might be caused by the weak dawn phase of the vascular enhancer (Figure 2b,c). However, we think the phase reported by the ETSLA lines is a true reflection of the phase *TOC1* and *PRR7* promoters in this tissue because the phase of the *CCR2* promoter in the vascular ETSLA lines was not shifted compared with *CCR2p:LUC*, which is phased in the evening similar to *TOC1* (Figure 5d)*.* Luciferase activity in the CCR2 mesophyll ETSLA lines was poorly rhythmic (Figure 5d). This might reflect expression of this circadian output in this tissue or could be due to silencing of any one of the three transgenes. The latter is a potential pitfall of introducing multiple transgenes, but this has not been a barrier for the majority of ETSLA lines. In summary, these data confirm the utility of the ETSLA system to study tissue‐specific circadian rhythms and confirm distinct circadian oscillator behaviour in different tissue types.

**Figure 6:**
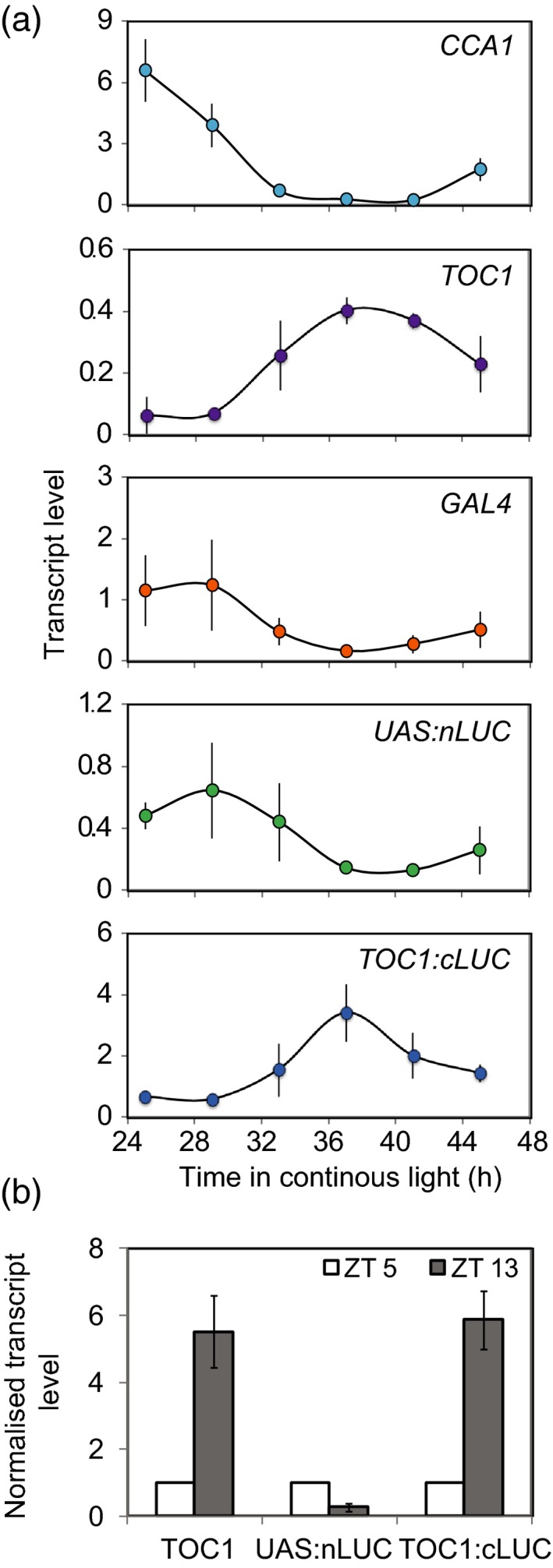
Transcript levels in the *TOC1* vascular enhancer trap split luciferase assay (ETSLA) line. Transcript levels of *TOC1, CCA1*, *GAL4, nLUC*, and *cLUC*, relative to *IPP1* and *PP2AA3* in 10–14‐day‐old *TOC1* vascular ETSLA line seedlings growing in (a) continuous light or (b) diel conditions. Values are means ± SD, *n* = 3.

Having observed striking phase difference of the *TOC1* promoter in the vascular ETSLA lines, we further examined the expression of transcripts in these lines by qRT‐PCR (Figure 6). We measured transcripts for oscillator genes and each of the three transgenes in shoots of F2 plants of the *TOC1* vascular ETSLA lines grown for 24 h in continuous light (Figure 6a). Robust rhythms of endogenous *CCA1* and *TOC1* expression were phased in the morning and evening, respectively, as expected. Robust, rhythmic expression was also detected for the *TOC1p:AC* transgene in the same phase as *TOC1,* confirming that the transgene is correctly expressed. Expression of *GAL4* and the transactivated *UAS:JN* transgene were similar in these plants to that observed for *GAL4* in the parental vascular enhancer trap line (Figure 3) with a low amplitude rhythm phased in the subjective morning. We also measured transcripts in these ETSLA F2 plants at ZT5 and ZT13 in a diel growth cycle (Figure 6b). We detected similar expression of the *TOC1p:AC* transgene to *TOC1* and low levels of expression of the transactivated *UAS:JN* transgene. Thus, in both diel and continuous light conditions, the relative expression of the dawn‐phased *nLUC* transcript is substantially lower than the expression of the evening‐phased *cLUC* transcript in the *TOC1* vascular ETSLA lines. This suggests that the phase shift of *TOC1* promoter activity in these lines is a true reflection of the phase of *TOC1* promoter activity in vascular tissue.

### Tissue‐specific expression of carbon starvation markers

**Figure 7:**
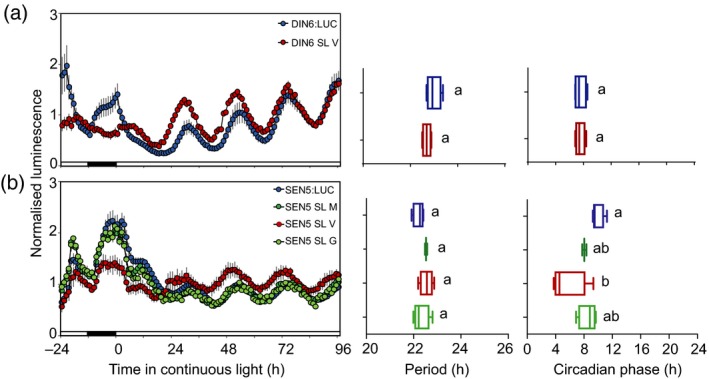
Tissue‐specific carbon starvation promoter activity using enhancer trap split luciferase assay (ETSLA). Normalized luminescence, period and phase estimates of luciferase activity in 10–14‐day‐old seedlings in continuous light. (a) *DIN6* vascular ETSLA line and (b) *SEN5* vascular, mesophyll, and guard cell ETSLA lines are shown, compared with the promoter:LUC control for each gene. Luminescence values are means ± SEM, *n* = 4. Period and phase estimates are represented as Min–Max box plots. Statistical differences from the promoter:LUC control were determined by one‐way anova with Tukey's post‐hoc tests. Different letters indicate significant differences between samples (*P* < 0.05).

Sugar signalling and metabolism are closely associated with the circadian clock (Blasing *et al.*, 2005; Graf *et al.*, 2010; Dalchau *et al.*, 2011; Haydon *et al.*, 2013; Haydon *et al.*, 2017) but little information is known about tissue‐specificity of sugar signalling networks. We generated promoter:AC constructs for *DARK INDUCIBLE 6 (DIN6)* and *SENESCENCE 5 (SEN5),* two transcriptional markers of Snf1‐related protein kinase 1 (SnRK1; Rodrigues *et al.*, 2013), which is a signalling hub for carbon starvation (Baena‐Gonzalez *et al.*, 2007) and modulator of circadian rhythms in Arabidopsis (Frank *et al.*, 2018). Robust circadian rhythms of luminescence were detected in both *DIN6p:LUC* and *SEN5p:LUC* transgenic lines (Figure 7), as expected (Frank *et al.*, 2018). No significant difference in circadian period or phase was detected between *DIN6p:LUC* and the *DIN6* vascular ETSLA line (Figure 7a). This suggests that, similar to *CCR2* (Figure 5d), morning phasing of this circadian output is not apparent in vascular tissue. For the *SEN5* promoter, we were able to isolate ETSLA lines for mesophyll, vasculature and guard cells (Figure 7b). Robust luciferase rhythms were detected in the *SEN5* guard cell ETSLA line, suggesting this system is compatible with detecting luciferase expression in potentially very small pools of cells. No difference in circadian period was detected between any ETSLA lines and the *SEN5p:LUC* control, but a significantly earlier phase was detected for the vascular line, similar to *TOC1* and *PRR7*. Thus, there might be heterogeneity in distinct networks of circadian outputs.

## Discussion

By building and verifying constructs for a split luciferase system that is compatible with *GAL4* enhancer trap lines, we have established the tools and demonstrated utility of a versatile transgenic toolset for spatiotemporal measurement of gene expression in Arabidopsis. We have demonstrated heterogeneity of gene expression dynamics of core circadian clock gene promoters and circadian‐regulated outputs. We have shown significant differences between the period and/or phase of circadian rhythms in vascular tissue compared with leaf mesophyll and whole‐seedling rhythms for promoters of *TOC1, PRR7*, and *SEN5*, but not *CCA1, CCR2*, or DIN6. This indicates that although there is spatial heterogeneity of circadian rhythms in Arabidopsis leaves, it is not representative of all circadian oscillator components or outputs, suggesting the existence of distinct circadian networks in particular cell types.

We have used the ETSLA system to measure expression of circadian‐regulated promoters because the spatial heterogeneity and organization of circadian oscillators in plant cells is emerging as a fascinating area of research (Endo *et al.*, 2014; Takahashi *et al.*, 2015; Gould *et al.*, 2018; Greenwood *et al.*, 2019). To ensure each enhancer trap line was suitable for this purpose, we measured circadian rhythms of the *GAL4* transcript, driven by the enhancer. This validation could be similarly achieved by introducing a *UAS:LUC* transgene. The tools we have generated are applicable to explore tissue‐specific dynamics of gene expression for any stimuli of interest. The particular advantages of the ETSLA system are that detection of luminescence is non‐destructive and can be measured in living plants growing in controlled conditions. Luminescence detection is not restricted by position of cell types within the tissue and has sufficient sensitivity to measure relatively small populations of cells. We have successfully detected tissue‐specific gene expression using enhancer trap lines for vasculature, mesophyll, and guard cells, but the system is compatible with numerous published lines (Laplaze *et al.*, 2005; Gardner *et al.*, 2009; Ckurshumova *et al.*, 2009; Radoeva *et al.*, 2016; Table S1) among hundreds available from seed stock centres. Once the lines have been generated, luminescence experiments can be easily performed. Thus, the ETSLA system is ideally suited to explore effects of environmental conditions or pharmacological treatments on gene expression. For circadian clock research, these approaches could provide insight into communication between oscillators in different cells.

The ETSLA system can be easily utilized by generating a single construct using the Gateway®‐compatible GW:AC plasmid. This can be used to introduce any promoter of interest and also may be suitable for translational fusions to the A‐FOS:cLUC fragment. Transformants can be crossed into any *GAL4 GFP* enhancer trap line carrying the *UAS:JN* transgene, which we have obtained for four lines in this study. This strategy of independent *AC* and *JN* transgenes allows for control of transgene position effects. However, introducing the *UAS:JN* sequence into the GW:AC plasmid might be worthwhile improvement to the ETSLA system.

We did not identify epidermal ETSLA lines with detectable luminescence in this study. Notwithstanding that we could show effective tissue‐specific expression of a *UAS:GUS* transgene (Figure 2a) and previously a *UAS:AEQUORIN* (Martí *et al.*, 2013) in the epidermal enhancer trap line, the lack of signal in the ETSLA lines might be due to large vacuoles in epidermal cells affecting luciferase signal or low promoter activity in these cells of the particular genes investigated. Epidermal *GAL4 GFP* lines with a *UAS:JN* transgene identified in this study could be used to test other promoter:AC constructs.

Our observation of distinct circadian rhythms in vascular tissue is consistent with a previous study (Endo *et al.*, 2014). A *SUC2p/TOC1p* TSLA line, which drives expression specifically in phloem companion cells was reported with a later phase compared with a *35Sp/TOC1p* TSLA line. We did not detect a significant difference in circadian rhythms of the *SUC2p/TOC1p* TSLA line in our experiments compared with *TOC1p:LUC* or *TOC1* mesophyll ETSLA seedlings. This may be due to the different control line or inclusion of sucrose in the media, which alters circadian rhythms in Arabidopsis (Haydon *et al.*, 2013;, 2017) and activity of the *SUC2* promoter (Truernit and Sauer, 1995). By contrast, we observed both significantly lengthened period and phase advance of *TOC1* promoter activity in vascular ETSLA lines, with similar effects for *PRR7* and *CCR2* promoters. The very different luciferase rhythms in *TOC1* vascular ETSLA lines compared with the *SUC2p/TOC1p* TSLA line is likely because of the different expression pattern of *GAL4* and *UAS:GUS* in the vascular enhancer trap line (KC274), for which we detected a much broader pattern of expression throughout vascular bundles compared with *SUC2* promoter activity specifically in phloem companion cells. Thus, there appears to be spatial heterogeneity of circadian rhythms even within vascular tissues.

The tissue‐specific features of circadian oscillators might be important for regulating distinct physiological or developmental outputs. Expression of *CCA1* from a range of tissue‐specific promoters resulted in different effects on photoperiodic flowering (Shimizu *et al.*, 2015). Expression of *CCA1* in phloem companion cells from the *SUC2* promoter delayed flowering in long days, but not when *CCA1* was expressed from *IRREGULAR XYLEM 3 (IRX3)* or *HOMEBOX GENE 8 (HB8)* promoters which are specific to xylem and procambium, respectively (Shimizu *et al.*, 2015). While these results are consistent with a specific role for the oscillator in companion cells for regulating flowering, they could also be explained by relatively high expression of *SUC2,* compared with *IRX3* and *HB8*, or different phases of the three promoters (Mockler *et al.*, 2007). Indeed, our characterization of the circadian expression of *GAL4* in each enhancer trap line demonstrates the importance of considering the expression dynamics of any chosen tissue‐specific promoter.

The differences in gene expression dynamics of circadian clock genes, which we detected in different ETSLA lines could be due to differences in light sensitivity of particular cell types. For example, the earlier light‐activated peak of *PRR7p:LUC* appeared more pronounced in the vascular ETSLA line compared with whole‐seedling rhythms. It has been suggested that light‐piping through vascular tissue might contribute to maintain circadian rhythms in Arabidopsis roots (Bordage *et al.*, 2016). Vascular cells might be more sensitive to light signals, or transmission of light through vasculature might be more efficient than mesophyll. The ETSLA system could be an effective tool to explore these dynamics in a wider range of vascular cell types, since several vascular *GAL4 GFP* enhancer trap lines are available (Table S1).

## Conclusion

We have adapted a split luciferase system to be compatible with available collections of enhancer trap lines and Gateway®‐compatible vectors to provide a versatile system for monitoring dynamic gene expression in specific tissues or cell types. We have validated a small selection of enhancer trap lines for leaf expression and confirmed their suitability to measure tissue‐specific circadian rhythms. We have corroborated previous data suggesting heterogeneity in circadian behaviour between leaf mesophyll and vascular tissues and expanded the tool set to investigate this behaviour. We hope this tool will provide a flexible resource to advance research to explore spatial heterogeneity in gene expression and identify sensitivity of particular tissues to various environmental stimuli and endogenous signals in intact, living plants.

## Experimental Procedures

### Plasmid constructs

TOC1p*:*AC and CCA1p:AC plasmids have been described (Endo *et al.*, 2014). To generate CCR2p:AC, a 2024 bp promoter fragment was amplified from gDNA of Columbia‐0 (Col‐0) by PCR with primers in containing *Hin*dIII sites and ligated into the TOC1p:AC plasmid in place of the *TOC1p* sequence. The GW:AC plasmid was made by PCR amplification of a 544 bp *A‐Fos:nLUC* (AC) fragment from TOC1p:AC using primers containing *Xba*I and *Spe*I sites and ligated into pEarlyGate301 (Earley *et al.*, 2006). For the *PRR7p:AC*, *SEN5p:AC* and *DIN6p:AC*, a 1020 bp *PRR7* promoter, 1666 bp *SEN5* promoter and 1017 bp *DIN6* were amplified by PCR from Col‐0 gDNA and cloned into pENTR/D‐TOPO (Invitrogen) and recombined into the with GW:AC plasmid. The *SEN5* promoter was also cloned into pEarleyGate301‐LUC+, comprised of a LUC+ fragment amplified from *CCR2p:LUC+* seedlings (Haydon *et al.*, 2013) and ligated into *Xba*I sites of pEarleyGate301 (Earley *et al.*, 2006).

The UAS:JN plasmid was generated by PCR amplification of a 656 bp c‐Jun:cLUC (JN) fragment from gDNA of TOC1:AC/SUC2:JN seedlings (Endo *et al.*, 2014) with primers containing *Bam*HI and *Sac*I restriction sites. The JN sequence was ligated into pBINYFPAEQ plasmid (Kiegle *et al.*, 2000) in place of the YFP:AEQ sequence, downstream of the UAS. The UAS:GUS plasmid has been described previously (Møller *et al.*, 2009).

All primers are listed in Table S3.

### Plant materials

Stable transgenic lines for *CCA1p:LUC+*, *TOC1p:LUC+*, *PRR7p:LUC+*, *CCR2p:LUC+*, *DIN6p:LUC+* and *TOC1p:AC/ SUC2p:JN* are in Col‐0 and have been used previously (Haydon *et al.*, 2013; Endo *et al.*, 2014; Frank *et al.*, 2018). The *GAL4 GFP* enhancer trap lines E1728, JR11‐2, KC274 and KC464 are in C24 and have been described (Gardner *et al.*, 2009; Martí *et al.*, 2013).

*TOC1p:AC, PRR7p:AC, CCA1p:AC, CCR2p:AC, SEN5p:AC* and *DIN6:AC* were transformed, or co‐transformed with *UAS:JN*, into Col‐0 by floral dip (Clough and Bent, 1998). *UAS:JN* was also transformed into each of the *GAL4 GFP* enhancer trap lines. Homozygous T3 populations of plants harbouring both the *promoter:AC* and *UAS:JN* transgenes were crossed to *GAL4 GFP* lines. As an alternative approach*, UAS:JN* transformants were identified for all four *GAL4 GFP* enhancer trap lines and T1‐T2 populations of *promoter:AC* transformants were crossed to T1 populations of *UAS:JN* transformants. Experiments were performed with F1 or F2 populations which would be heterozygous or segregating for the three dominant transgenes, respectively.

### Luminescence experiments

Seeds were surface sterilized with a solution of 20% (v/v) bleach, 0.02% (v/v) Triton X‐100 and washed three times with sterile deionized water. Seeds were sown in clusters of five to twenty on modified Hoagland medium (Haydon *et al.*, 2012), solidified with 0.8% agar type M. Plates were chilled for 2 days at 4°C and grown in 12 h light, 12 h dark (LD) cycles at 20°C. Light was supplied from red (660 nm), green (550 nm), blue (450 nm) and far‐red (730 nm) LED arrays (HiPoint, Kaohsiung City, Taiwan) at 50 µmol m^−2^ sec^−1^. Here, 10–14‐day‐old seedlings were treated twice with a topical application of 1 mm d‐luciferin, K^+^ salt at least 24 h before photon counting. Luminescence was measured for 600 sec, following a 2 min delay to decay chlorophyll fluorescence (Gould *et al.*, 2009) at 1 h intervals for 48 h in LD and 120 h of continuous light in a HRPCS2 (Photek, St Leonards on Sea, East Sussex, UK) with light supplied from red (660 nm) and blue (470 nm) LEDs at 50 μmol m^−2^ sec^−1^. Luminescence for each cluster was normalized to average counts across the time series. Circadian period and circadian phase (corrected for circadian period in free‐running conditions) estimates were performed on raw luminescence data between 24 and 120 h in continuous light using fast Fourier Transform‐nonlinear least squares (FFT‐NLLS) analysis, using BioDare2 (https://biodare2.ed.ac.uk/) (Zielinski *et al.*, 2014). Spatial imaging of luminescence in ETSLA lines (Figure 4) used a NightShade LB 985 Plant Imaging System (Berthold, Bad Wildbad, Germany).

### *Thermal asymmetric interlaced *(*TAIL*)* PCR*

The genomic locations of the *GAL4 GFP* T‐DNAs were determined by TAIL PCR, essentially as described (Liu and Whittier, 1995), using nested specific primers complementary to the right or left T‐DNA borders and a degenerate primer (Table S3), as described (Gardner *et al.*, 2009). The products of the tertiary reaction were cloned and sequenced to identify the flanking genomic region(s) of the T‐DNA.

### Quantitative RT‐PCR

Shoots of 14‐day‐old seedlings were snap frozen in liquid N. Total RNA was extracted from frozen tissue using Isolate II Plant RNA Kit (Bioline) with on‐column DNase I treatment. cDNA was prepared from 0.5 μg RNA with Tetro cDNA Synthesis Kit (Bioline, Alexandria, NSW, Australia) using oligo‐dT primer. Technical replicates of gene‐specific products were amplified with primers in Table S2 in 10 μl reactions using SensiFAST SYBR no‐ROX kit (Bioline) on a CFX96 Thermocycler (Bio‐Rad). Transcript levels were calculated from C_t_ values, incorporating PCR efficiencies calculated with LinRegPCR (Ruijter *et al.*, 2009), relative to the geometric mean of two reference genes *ISOPENTENYL PYROPHOSPHATE:DIMETHYLALLYL PYROPHOSPHATE ISOMERASE 2 (IPP2)* and *PROTEIN PHOSPHATASE 2A SUBUNIT A3 (PP2AA3)* (Vandesompele *et al.*, 2002).

### β‐Glucuronidase (GUS) stains

T1 seedlings of *GAL4 GFP* enhancer trap lines transformed with UAS:GUS were GUS‐stained overnight as previously (Haydon and Cobbett, 2007) and imaged with a SMZ800 stereomicroscope (Nikon, Rhodes, NSW, Australia). For leaf sections, seedlings were fixed in a formaldehyde:acetic acid:ethanol (3.7%:5%:50%) mix, dehydrated in a series of an increasingly concentrated ethanol solution and imbibed in a series of Histoclear reagent. Tissue was infiltrated with wax (Paraplast plus, St Louis, MO, USA) and subsequently sectioned (8 µm). Sections were imaged using a BX60 microscope (Olympus, Notting Hill, VIC, Australia) and differential interference contrast (DIC) optics.

## Author Contributions

MH and AW conceived the study, AR and MH designed experiments, AR, JG, and MH performed experiments, all authors analyzed and/or interpreted data, AR and MH drafted the manuscript, all authors revised and approved the manuscript.

## Supporting information


**Table S1.** List of published GAL4 enhancer trap lines compatible with the ETSLA system.
**Table S2.** Summary of transgenic lines used in this study.
**Table S3.** Sequences of primers used in this study.

## Data Availability

All data generated and used in this study are available upon request or as Supporting Information for this article.

## References

[tpj14603-bib-0001] Asano, T. , Masumura, T. , Kusano, H. , Kikuchi, S. , Kurita, A. , Shimada, H. and Kadowaki, K.‐I. (2002) Construction of a specialized cDNA library from plant cells isolated by laser capture microdissection: toward comprehensive analysis of the genes expressed in the rice phloem. Plant J. 32, 401–408. 12410817 10.1046/j.1365-313x.2002.01423.x

[tpj14603-bib-0002] Baena‐Gonzalez, E. , Rolland, F. , Thevelein, J.M. and Sheen, J. (2007) A central integrator of transcription networks in plant stress and energy signalling. Nature, 448, 938–942. 17671505 10.1038/nature06069

[tpj14603-bib-0003] Birnbaum, K. , Shasha, D.E. , Wang, J.Y. , Jung, J.W. , Lambert, G.M. , Galbraith, D.W. and Benfey, P.N. (2003) A gene expression map of the Arabidopsis root. Science, 302, 1956–1960. 14671301 10.1126/science.1090022

[tpj14603-bib-0004] Blasing, O.E. , Gibon, Y. , Gunther, M. ***et al*** **.** (2005) Sugars and circadian regulation make major contributions to the global regulation of diurnal gene expression in Arabidopsis. Plant Cell, 17, 3257–3281. 16299223 10.1105/tpc.105.035261 PMC1315368

[tpj14603-bib-0005] Bonner, R.F. , Emmert‐Buck, M. , Cole, K. , Pohida, T. , Chuaqui, R. , Goldstein, S. and Liotta, L.A. (1997) Laser capture microdissection: molecular analysis of tissue. Science, 278, 1481–1483. 9411767 10.1126/science.278.5342.1481

[tpj14603-bib-0006] Bordage, S. , Sullivan, S. , Laird, J. , Millar, A.J. and Nimmo, H.G. (2016) Organ specificity in the plant circadian system is explained by different light inputs to the shoot and root clocks. New Phytol. 212, 136–149. 27240972 10.1111/nph.14024 PMC5006879

[tpj14603-bib-0007] Brady, S.M. , Song, S. , Dhugga, K.S. , Rafalski, J.A. and Benfey, P.N. (2007) Combining expression and comparative evolutionary analysis. The COBRA gene family. Plant Physiol. 143, 172–187. 17098858 10.1104/pp.106.087262 PMC1761980

[tpj14603-bib-0008] Ckurshumova, W. , Koizumi, K. , Chatfield, S.P. , Sanchez‐Buelna, S.U. , Gangaeva, A.E. , McKenzie, R. and Berleth, T. (2009) Tissue‐specific GAL4 expression patterns as a resource enabling targeted gene expression, cell type‐specific transcript profiling and gene function characterization in the Arabidopsis vascular system. Plant Cell Physiol. 50, 141–150. 19068493 10.1093/pcp/pcn180

[tpj14603-bib-0009] Clough, S.J. and Bent, A.F. (1998) Floral dip: A simplified method for *Agrobacterium*‐mediated transformation of *Arabidopsis thaliana* . Plant J. 16, 735–743. 10069079 10.1046/j.1365-313x.1998.00343.x

[tpj14603-bib-0010] Coker, T.L.R. , Cevik, V. , Beynon, J.L. and Gifford, M.L. (2015) Spatial dissection of the *Arabidopsis thaliana* transcriptional response to downy mildew using fluorescence activated cell sorting. Front. Plant Sci. 6, 1–13. 26217372 10.3389/fpls.2015.00527 PMC4498041

[tpj14603-bib-0011] Dalchau, N. , Baek, S.J. , Briggs, H.M. ***et al*** **.** (2011) The circadian oscillator gene GIGANTEA mediates a long‐term response of the *Arabidopsis thaliana* circadian clock to sucrose. Proc. Natl. Acad. Sci. USA, 108, 5104–5109. 21383174 10.1073/pnas.1015452108 PMC3064355

[tpj14603-bib-0012] Deal, R.B. and Henikoff, S. (2011) The INTACT method for cell type‐specific gene expression and chromatin profiling in *Arabidopsis thaliana* . Nat. Protoc. 6, 56–68. 21212783 10.1038/nprot.2010.175 PMC7219316

[tpj14603-bib-0013] Del Toro‐De Leon, G. and Kohler, C. (2018) Endosperm‐specific transcriptome analysis by applying the INTACT system. Plant Reprod. 32, 55–61. 30588542 10.1007/s00497-018-00356-3

[tpj14603-bib-0014] Denyer, T. , Ma, X. , Klesen, S. , Scacchi, E. , Nieselt, K. and Timmermans, M.C.P. (2019) Spatiotemporal developmental trajectories in the Arabidopsis root revealed using high‐throughput single‐cell RNA Sequencing. Dev. Cell, 48, 840–852. 30913408 10.1016/j.devcel.2019.02.022

[tpj14603-bib-0015] Dinneny, J.R. , Long, T.A. , Wang, J.Y. ***et al*** **.** (2008) Cell identity mediates the response of Arabidopsis roots to abiotic stress. Science, 320, 942–945. 18436742 10.1126/science.1153795

[tpj14603-bib-0016] Dodd, A.N. , Jakobsen, M.K. , Baker, A.J. ***et al*** **.** (2006) Time of day modulates low‐temperature Ca2+ signals in Arabidopsis. Plant J. 48, 962–973. 17227550 10.1111/j.1365-313X.2006.02933.x

[tpj14603-bib-0017] Earley, K.W. , Haag, J.R. , Pontes, O. , Opper, K. , Juehne, T. , Song, K. and Pikaard, C.S. (2006) Gateway‐compatible vectors for plant functional genomics and proteomics. Plant J. 45, 616–629. 16441352 10.1111/j.1365-313X.2005.02617.x

[tpj14603-bib-0018] Efroni, I. and Birnbaum, K.D. (2016) The potential of single‐cell profiling in plants. Genome Biol. 17, 1–8. 27048384 10.1186/s13059-016-0931-2 PMC4820866

[tpj14603-bib-0019] Emmert‐Buck, M.R. , Bonner, R.F. , Smith, P.D. , Chuaqui, R.F. , Zhuang, Z. , Goldstein, S.R. , Weiss, R.A. and Liotta, L.A. (1996) Laser capture microdissection. Science, 274, 998–1001. 8875945 10.1126/science.274.5289.998

[tpj14603-bib-0020] Endo, M. , Shimizu, H. , Nohales, M.A. , Araki, T. and Kay, S.A. (2014) Tissue‐specific clocks in Arabidopsis show asymmetric coupling. Nature, 515, 419–422. 25363766 10.1038/nature13919 PMC4270698

[tpj14603-bib-0021] Frank, A. , Matiolli, C.C. , Viana, A.J.C. ***et al*** **.** (2018) Circadian entrainment in Arabidopsis by the sugar‐responsive transcription factor bZIP63. Curr. Biol. 28, 2597–2606. 30078562 10.1016/j.cub.2018.05.092 PMC6108399

[tpj14603-bib-0022] Gan, Y. , Bernreiter, A. , Filleur, S. , Abram, B. and Forde, B.G. (2012) Overexpressing the ANR1 MADS‐box gene in transgenic plants provides new insights into its role in the nitrate regulation of root development. Plant Cell Physiol. 53, 1003–1016. 22523192 10.1093/pcp/pcs050

[tpj14603-bib-0023] Gardner, M.J. , Baker, A.J. , Assie, J.‐M.M. , Poethig, R.S. , Haseloff, J.P. and Webb, A.A.R. (2009) GAL4 GFP enhancer trap lines for analysis of stomatal guard cell development and gene expression. J. Exp. Bot. 60, 213–226. 19033548 10.1093/jxb/ern292 PMC3071773

[tpj14603-bib-0024] Gould, P.D. , Diaz, P. , Hogben, C. , Kusakina, J. , Salem, R. , Hartwell, J. and Hall, A. (2009) Delayed fluorescence as a universal tool for the measurement of circadian rhythms in higher plants. Plant J. 58, 893–901. 19638147 10.1111/j.1365-313X.2009.03819.x

[tpj14603-bib-0025] Gould, P.D. , Domijan, M. , Greenwood, M. , Tokuda, I.T. , Rees, H. , Kozma‐Bognar, L. , Hall, A.J. and Locke, J.C. (2018) Coordination of robust single cell rhythms in the Arabidopsis circadian clock via spatial waves of gene expression. Elife, 7, 1–20. 10.7554/eLife.31700 PMC5988422 29697372

[tpj14603-bib-0026] Graf, A. , Schlereth, A. , Stitt, M. and Smith, A.M. (2010) Circadian control of carbohydrate availability for growth in Arabidopsis plants at night. Proc. Natl. Acad. Sci. USA, 107, 9458–9463. 20439704 10.1073/pnas.0914299107 PMC2889127

[tpj14603-bib-0027] Greenwood, M. , Domijan, M. , Gould, P.D. , Hall, A.J.W. and Locke, J.C.W. (2019) Coordinated circadian timing through the integration of local inputs in *Arabidopsis thaliana* . PLoS Biol. 17(8), e3000407 10.1371/journal.pbio.3000407. 31415556 PMC6695092

[tpj14603-bib-0028] Grønlund, J.T. , Eyres, A. , Kumar, S. , Buchanan‐Wollaston, V. and Gifford, M.L. (2012) Cell specific analysis of Arabidopsis leaves using fluorescence activated cell sorting. J. Vis. Exp. 68, 1–6. 10.3791/4214 PMC3490320 23070217

[tpj14603-bib-0029] Haseloff, J. (1999) GFP variants for multispectral imaging of living cells. Methods Cell Biol. 58, 139–151. 9891379 10.1016/s0091-679x(08)61953-6

[tpj14603-bib-0030] Haydon, M.J. and Cobbett, C.S. (2007) A novel major facilitator superfamily protein at the tonoplast influences zinc tolerance and accumulation in Arabidopsis. Plant Physiol. 143, 1705–1719. 17277087 10.1104/pp.106.092015 PMC1851824

[tpj14603-bib-0031] Haydon, M.J. , Kawachi, M. , Wirtz, M. , Hillmer, S. , Hell, R. and Kramer, U. (2012) Vacuolar nicotianamine has critical and distinct roles under iron deficiency and for zinc sequestration in Arabidopsis. Plant Cell, 24, 724–737. 22374397 10.1105/tpc.111.095042 PMC3315243

[tpj14603-bib-0032] Haydon, M.J. , Mielczarek, O. , Robertson, F.C. , Hubbard, K.E. and Webb, A.A.R.R. (2013) Photosynthetic entrainment of the *Arabidopsis thaliana* circadian clock. Nature, 502, 689–692. 24153186 10.1038/nature12603 PMC3827739

[tpj14603-bib-0033] Haydon, M.J. , Mielczarek, O. , Frank, A. , Roman, A. and Webb, A.A.R. (2017) Sucrose and ethylene signaling interact to modulate the circadian clock. Plant Physiol. 175, 947–958. 28778922 10.1104/pp.17.00592 PMC5619894

[tpj14603-bib-0034] Haydon, M.J. , Li, X. and Ting, M.K.‐Y. (2019) Temporal control of plant‐environment interactions by the circadian clock. Annu. Plant Rev. 2, 1–32.

[tpj14603-bib-0035] James, A.B. , Monreal, J.A. , Nimmo, G.A. , Kelly, C.L. , Herzyk, P. , Jenkins, G.I. and Nimmo, H.G. (2008) The circadian clock in Arabidopsis roots is a simplified slave version of the clock in shoots. Science, 322, 1832–1835. 19095940 10.1126/science.1161403

[tpj14603-bib-0036] Jean‐Baptiste, K. , McFaline‐Figueroa, J.L. , Alexandre, C.M. ***et al*** **.** (2019) Dynamics of gene expression in single root cells of *Arabidopsis thaliana* . Plant Cell, 31, 993–1011. 30923229 10.1105/tpc.18.00785 PMC8516002

[tpj14603-bib-0037] Jia, H. , Loock, B.Van , Liao, M. , Verbelen, J.‐P. and Vissenberg, K. (2007) Combination of the ALCR/alcA ethanol switch and GAL4/VP16‐UAS enhancer trap system enables spatial and temporal control of transgene expression in Arabidopsis. Plant Biotechnol. J. 5, 477–482. 17442066 10.1111/j.1467-7652.2007.00255.x

[tpj14603-bib-0038] Kerk, N.M. , Ceserani, T. , Tausta, S.L. , Sussex, I.M. and Nelson, T.M. (2003) Laser capture microdissection of cells from plant tissues. Plant Physiol. 132, 27–35. 12746508 10.1104/pp.102.018127 PMC1540312

[tpj14603-bib-0039] Kiegle, E. , Moore, C.A. , Haseloff, J. , Tester, M.A. and Knight, M.R. (2000) Cell‐type‐specific calcium responses to drought, salt and cold in the Arabidopsis root. Plant J. 23, 267–278. 10929120 10.1046/j.1365-313x.2000.00786.x

[tpj14603-bib-0040] Kim, H. , Kim, Y. , Yeom, M. , Lim, J. and Nam, H.G. (2016) Age‐associated circadian period changes in Arabidopsis leaves. J. Exp. Bot. 67, 2665–2673. 27012281 10.1093/jxb/erw097 PMC4861015

[tpj14603-bib-0041] Laplaze, L. , Parizot, B. , Baker, A. ***et al*** **.** (2005) GAL4‐GFP enhancer trap lines for genetic manipulation of lateral root development in *Arabidopsis thaliana* . J. Exp. Bot. 56, 2433–2442. 16043452 10.1093/jxb/eri236

[tpj14603-bib-0042] Laplaze, L. , Benkova, E. , Casimiro, I. ***et al*** **.** (2007) Cytokinins act directly on lateral root founder cells to inhibit root initiation. Plant Cell, 19, 3889–3900. 18065686 10.1105/tpc.107.055863 PMC2217640

[tpj14603-bib-0043] Liu, Y.‐G. and Whittier, R.F. (1995) Thermal asymmetric interlaced PCR: automatable amplification and sequencing of insert end fragments from P1 and YAC clones for chromosome walking. Genomics, 25, 674–681. 7759102 10.1016/0888-7543(95)80010-j

[tpj14603-bib-0044] Martí, M.C. , Stancombe, M.A. and Webb, A.A.R. (2013) Cell‐ and stimulus type‐specific intracellular free Ca2+ signals in Arabidopsis. Plant Physiol. 163, 625–634. 24027243 10.1104/pp.113.222901 PMC3793043

[tpj14603-bib-0045] Mockler, T. , Michael, T. , Priest, H. , Shen, R. , Sullivan, C. , Givan, S. , McEntee, C. , Kay, S. and Chory, J. (2007) THE DIURNAL PROJECT: Diurnal and circadian expression profiling, model‐based pattern matching and promoter analysis. Cold Spring Harb. Symp. Quant. Biol. 72, 353–363. 18419293 10.1101/sqb.2007.72.006

[tpj14603-bib-0046] Møller, I.S. , Gilliham, M. , Jha, D. , Mayo, G.M. , Roy, S.J. , Coates, J.C. , Haseloff, J. and Tester, M. (2009) Shoot Na^+^ exclusion and increased salinity tolerance engineered by cell type–specific alteration of Na^+^ transport in Arabidopsis. Plant Cell, 21, 2163–2178. 19584143 10.1105/tpc.108.064568 PMC2729596

[tpj14603-bib-0047] Moreno‐Romero, J. , Santos‐Gonzalez, J. , Hennig, L. and Kohler, C. (2017) Applying the INTACT method to purify endosperm nuclei and to generate parental‐specific epigenome profiles. Nat. Protoc. 12, 238–254. 28055034 10.1038/nprot.2016.167

[tpj14603-bib-0048] Nakazono, M. , Qiu, F. , Borsuk, L.A. and Schnable, P.S. (2003) Laser‐capture microdissection, a tool for the global analysis of gene expression in specific plant cell types: identification of genes expressed differentially in epidermal cells or vascular tissues of maize. Plant Cell, 15, 583–96. 12615934 10.1105/tpc.008102 PMC150015

[tpj14603-bib-0049] Para, A. , Farre, E.M. , Imaizumi, T. , Pruneda‐Paz, J.L. , Harmon, F.G. and Kay, S.A. (2007) PRR3 Is a vascular regulator of TOC1 stability in the Arabidopsis circadian clock. Plant Cell, 19, 3462–3473. 18055606 10.1105/tpc.107.054775 PMC2174887

[tpj14603-bib-0050] Radoeva, T. , Hove, C.A.Ten , Saiga, S. and Weijers, D. (2016) Molecular characterization of Arabidopsis GAL4/UAS enhancer trap lines identifies novel cell‐type‐specific promoters. Plant Physiol. 171, 1169–1181. 27208300 10.1104/pp.16.00213 PMC4902605

[tpj14603-bib-0051] Reynoso, M.A. , Pauluzzi, G.C. , Kajala, K. ***et al*** **.** (2018) Nuclear transcriptomes at high resolution using retooled INTACT. Plant Physiol. 176, 270–281. 28956755 10.1104/pp.17.00688 PMC5761756

[tpj14603-bib-0052] Rodrigues, A. , Adamo, M. , Crozet, P. ***et al*** **.** (2013) ABI1 and PP2CA phosphatases are negative regulators of Snf1‐related protein kinase1 signaling in Arabidopsis. Plant Cell, 25, 3871–3884. 24179127 10.1105/tpc.113.114066 PMC3877788

[tpj14603-bib-0053] Ron, M. , Kajala, K. , Pauluzzi, G. ***et al*** **.** (2014) Hairy root transformation using *Agrobacterium* rhizogenes as a tool for exploring cell type‐specific gene expression and function using tomato as a model. Plant Physiol. 166, 455–469. 24868032 10.1104/pp.114.239392 PMC4213079

[tpj14603-bib-0054] Ruijter, J.M. , Ramakers, C. , Hoogaars, W.M.H. , Karlen, Y. , Bakker, O. , van den Hoff, M.J.B. and Moorman, A.F.M. (2009) Amplification efficiency: linking baseline and bias in the analysis of quantitative PCR data. Nucleic Acids Res. 37, 1–12. 19237396 10.1093/nar/gkp045 PMC2665230

[tpj14603-bib-0055] Ryu, K.H. , Huang, L. , Kang, H.M. and Schiefelbein, J. (2019) Single‐cell RNA sequencing resolves molecular relationships among individual plant cells. Plant Physiol. 179, 1444–1456. 30718350 10.1104/pp.18.01482 PMC6446759

[tpj14603-bib-0056] Schulze, W.X. , Reinders, A. , Ward, J. , Lalonde, S. and Frommer, W.B. (2003) Interactions between co‐expressed Arabidopsis sucrose transporters in the split‐ubiquitin system. BMC Biochem. 4, 1–10. 12689351 10.1186/1471-2091-4-3 PMC153512

[tpj14603-bib-0057] Shimizu, H. , Katayama, K. , Koto, T. , Torii, K. , Araki, T. and Endo, M. (2015) Decentralized circadian clocks process thermal and photoperiodic cues in specific tissues. Nat. plants, 1, 1–7. 10.1038/nplants.2015.163 27251534

[tpj14603-bib-0058] Takahashi, N. , Hirata, Y. , Aihara, K. and Mas, P. (2015) A hierarchical multi‐oscillator network orchestrates the Arabidopsis circadian system. Cell, 163, 148–159. 26406375 10.1016/j.cell.2015.08.062

[tpj14603-bib-0059] Truernit, E. and Sauer, N. (1995) The promoter of the *Arabidopsis thaliana* SUC2 sucrose‐H+ symporter gene directs expression of beta‐glucuronidase to the phloem: evidence for phloem loading and unloading by SUC2. Planta, 196, 564–570. 7647685 10.1007/BF00203657

[tpj14603-bib-0060] Vandesompele, J. , De Preter, K. , Pattyn, F. , Poppe, B. , Van Roy, N. , De Paepe, A. and Speleman, F. (2002) Accurate normalization of real‐time quantitative RT‐PCR data by geometric averaging of multiple internal control genes. Genome Biol. 3, 1–12. 10.1186/gb-2002-3-7-research0034 PMC126239 12184808

[tpj14603-bib-0061] Wenden, B. , Toner, D.L.K. , Hodge, S.K. , Grima, R. and Millar, A.J. (2012) Spontaneous spatiotemporal waves of gene expression from biological clocks in the leaf. Proc. Natl. Acad. Sci. USA, 109, 6757–6762. 22496591 10.1073/pnas.1118814109 PMC3340055

[tpj14603-bib-0062] Xu, X. , Hotta, C.T. , Dodd, A.N. , Love, J. , Sharrock, R. , Lee, Y.W. , Xie, Q. , Johnson, C.H. and Webb, A.A.R.R. (2007) Distinct light and clock modulation of cytosolic free Ca2+ oscillations and rhythmic CHLOROPHYLL A/B BINDING PROTEIN2 promoter activity in Arabidopsis. Plant Cell, 19, 3474–3490. 17982000 10.1105/tpc.106.046011 PMC2174886

[tpj14603-bib-0063] Zielinski, T. , Moore, A.M. , Troup, E. , Halliday, K.J. and Millar, A.J. (2014) Strengths and limitations of period estimation methods for circadian data. PLoS ONE, 9, 1–26. 10.1371/journal.pone.0096462 PMC4014635 24809473

