# Supplementary material for: Combining GAL4 GFP enhancer trap with split luciferase to measure spatiotemporal promoter activity in Arabidopsis
Source: Plant J. 2019 Dec 3;102(1):187–98. doi: 10.1111/tpj.14603 (PMC7217008; doi:10.1111/tpj.14603)
Supplement: Supplementary file 1 — Table S1. List of published GAL4 enhancer trap lines compatible with the ETSLA system. Table S2. Summary of transgenic lines used in this study. Table S3. Sequences of primers used in this study. [file TPJ-102-187-s001.docx]

**Table S1.** List of published GAL4 enhancer trap lines compatible with the ETSLA system

| **Line/ABRC number** | **Expression Pattern** | | **References** |
| --- | --- | --- | --- |
|  | **Root** | **Shoot** |  |
| J0121/N9090 | Xylem pole pericycle cells | Young leaf primordia | Laplaze et al., 2005; Radoeva *et al*., 2016 |
| J0192 | Lateral root primordia |  | Laplaze *et al*., 2005 |
| J2772 | Root cap, lateral root primordia, base of lateral roots |  | Laplaze *et al*., 2005 |
| Q850 | Root cap, lateral root primordia |  | Laplaze *et al*., 2005 |
| N9113 |  | All tissues | Jia *et al*., 2007 |
| N9093 | Distal root and root-hypocotyl junction |  | Jia *et al*., 2007 |
| N9195 | Some specific tissues of the root | Leaves | Jia *et al*., 2007 |
| E1728 | nd | Guard cells | Dodd *et al*., 2006; Gardner *et al*., 2009 |
| E2036 | nd | Guard cells | Gardner *et al*., 2009 |
| E292 | nd | Guard cells | Gardner *et al*., 2009 |
| E361 | Epidermis, cortex | Guard cells, epidermis, mesophyll, cortex, apical meristem | Gardner *et al*., 2009 |
| E551 | nd | Guard cells, trichomes, epidermis | Gardner *et al*., 2009 |
| E566 | nd | Guard cells, trichomes, apical meristem | Gardner *et al*., 2009 |
| E910 | nd | Guard cells, trichomes, epidermis | Gardner *et al*., 2009 |
| E994 | Epidermis, vasculature, root tip | Guard cells, apical meristem | Gardner *et al*., 2009 |
| J1512 | Cortex | Guard cells | Gardner *et al*., 2009 |
| KS019 | Epidermis | Guard cells, apical meristem | Gardner *et al*., 2009 |
| Q1621 | Epidermis, vasculature | Guard cells, apical meristem | Gardner *et al*., 2009 |
| Q1622 | Vasculature | Guard cells | Gardner *et al*., 2009 |
| Q2480 | Epidermis, cortex, root tip | Guard cells | Gardner *et al*., 2009 |
| Q2481 | Epidermis, cortex, root tip | Guard cells | Gardner *et al*., 2009 |
| J2103 | Epidermis, vasculature, root tip | Guard cells, epidermis | Gardner *et al*., 2009 |
| R010/11 | Epidermis | Epidermis | Gardner *et al*., 2009 |
| Q2610 | All root tissues |  | Waki *et al*., 2013 |
| Q0990/N9217 | Vasculature | nd | Radoeva *et al*., 2016 |
| Q2500/N9135 | Ground tissue, pericycle and QC | Cotyledon junction and guard mother cell | Radoeva *et al*., 2016 |
| J0571/N9094 | Ground tissue, some QC cells | Guard and pavement cells | Radoeva *et al*., 2016 |
| J2731 | Cells above QC | Guard and pavement cells | Radoeva *et al*., 2016 |
| M0148/N9303 | Pericycle cells | Cells surrounding SAM | Radoeva *et al*., 2016 |
| M0164/N9307 | Pericycle | Shoot vasculature | Radoeva *et al*., 2016 |
| M0167/N9308 | nd | Cotyledon junction | Cary *et al.*, 2002; Radoeva *et al*., 2016 |
| M0223/N9336 | nd | Cotyledon junction | Cary *et al*., 2002; Radoeva *et al*., 2016 |
| J1092/N9147 | Root aplical meristem, LRC | nd | Radoeva *et al*., 2016 |
| J3281/N9128 | Young Vasculature tissue, columella cells | nd | Radoeva *et al*., 2016 |
| J3411/N9131 | Root aplical meristem, LRC | nd | Radoeva *et al*., 2016 |
| M0028/N9274 | Columella cells | nd | Radoeva *et al*., 2016 |
| Q0680/N9209 | Columella cells | nd | Radoeva *et al*., 2016 |
| Q1630/N9227 | Columella cells | nd | Radoeva *et al*., 2016 |
| Q0171/N9207 | Root cap | nd | Radoeva *et al*., 2016 |
| RM1000 | Leaf margins | nd | Radoeva *et al*., 2016 |
| J2501/N9121 | nd | nd | Radoeva *et al*., 2016 |
| J2661/N9187 | nd | nd | Radoeva *et al*., 2016 |
| M0136/N9302 | nd | nd | Radoeva *et al*., 2016 |
| M0171 |  | Suspensor cells | Radoeva *et al*., 2019 |

nd: not detected

**Table S2.** Summary of transgenic lines used in this study.

| **ETSLA line** | **Parent 1** | **Parent 2** | **Period**  **(mean ± SD)** | **Phase**  **(mean ± SD)** | **RAE**  **(mean ± SD)** | **Experiment** |
| --- | --- | --- | --- | --- | --- | --- |
| CCA1p:LUC | n/a | n/a | 22.2 ± 1.1 | 4.8 ± 0.79 | 0.31 ± 0.07 | Fig 4a |
| CCA1p:LUC | n/a | n/a | 22.3 ± 0.77 | 3.7 ± 1.0 | 0.25 ± 0.16 | - |
| CCA1p-V-LUC F1 | CCA1p:AC/UAS:JN T3 (A3850/52) | KC274 | 21.6 ± 0.08 | 5.6 ± 0.33 | 0.29 ± 0.02 | Fig 4a |
| CCA1p-V-LUC F1 | CCA1p:AC T1 (A3420) | UAS:JN/KC274 T1 (A3727) | 22.2 ± 0.19 | 5.0 ± 0.92 | 0.33 ± 0.13 | - |
| CCA1p-M-LUC F1 | CCA1p:AC/UAS:JN T3 (A3850/52) | JR11-2 | 21.6 ± 0.18 | 5.2 ± 0.63 | 0.41 ± 0.07 | Fig 4a |
| CCA1p-M-LUC F1 | CCA1p:AC T1 (A3420) | UAS:JN/JR11-2 T1 (A3740) | 22.0 ± 0.17 | 6.7 ± 0.45* | 0.70 ± 0.15 | - |
| PRR7p:LUC | n/a | n/a | 20.5 ± 0.28 | 13.8 ± 2.2 | 0.27 ± 0.05 | Fig 4b |
| PRR7p:LUC | n/a | n/a | 21.5 ± 1.0 | 14.2 ± 2.5 | 0.13 ± 0.05 | - |
| PRR7p-V-LUC F1 | PRR7p:AC  T1 (A3922) | UAS:JN/KC274 T1 (A3727) | 22.6 ± 0.29 | 5.3 ± 0.63* | 0.24 ± 0.04 | Fig 4b |
| PRR7p-V-LUC F1 | PRR7p:AC  T2 (A3922) | UAS:JN/KC274 T1 (A3738) | 22.4 ± 0.29* | 8.5 ± 1.1* | 0.28 ± 0.12 | - |
| PRR7p-M-LUC F1 | PRR7p:AC  T1 (A3922) | UAS:JN/JR11-2 T1 (A3743) | 21.3 ± 0.34* | 12.8 ± 4.5 | 0.42 ± 0.10 | Fig 4b |
| PRR7p-M-LUC F1 | PRR7p:AC  T2 (A3922/4112) | UAS:JN/JR11-2 T1 (A3740) | 23.4 ± 1.1 | 12.9 ± 0.21 | 0.37 ± 0.17 | - |
| TOC1p:LUC | n/a | n/a | 22.8 ± 0.19 | 15.3 ±0.26 | 0.08 ± 0.01 | Fig 4c |
| TOC1p:LUC | n/a | n/a | 22.6 ± 0.38 | 17.3 ± 0.85 | 0.18 ± 0.06 | - |
| TOC1/SUC2 TSLA | n/a | n/a | 22.0 ± 0.14 | 15.6 ± 0.86 | 0.17 ±0.02 | Fig 4c |
| TOC1p-V-LUC F2 (A5073) | TOC1p:AC/UAS:JN T3 (A3452) | KC274 | 23.7 ± 0.99* | 3.2 ± 0.58* | 0.28 ± 0.11 | Fig 4c |
| TOC1p-V-LUC F1 | TOC1p:AC/UAS:JN T3 (A3942/49) | KC274 | 22.2 ± 0.73 | 7.8 ± 3.2* | 0.36 ± 0.10 | - |
| TOC1p-M-LUC F2 (A5078) | TOC1p:AC/UAS:JN T3 (A3452) | JR11-2 | 23.2 ± 0.26 | 16.6 ± 0.66* | 0.15 ± 0.03 | Fig 4c |
| TOC1p-M-LUC F1 | TOC1p:AC/UAS:JN T3 (A3942/49) | JR11-2 | 23.4 ± 1.2 | 14.1 ± 3.8 | 0.52 ± 0.24 | - |
| CCR2p:LUC | n/a | n/a | 22.0 ± 1.2 | 14.8 ± 2.5 | 0.28 ± 0.04 | Fig 4d |
| CCR2p:LUC | n/a | n/a | 22.9 ± 0.77 | 14.5 ± 0.99 | 0.22 ± 0.04 | - |
| CCR2p-V-LUC F1 | CCR2p:AC  T1 (A3433) | UAS:JN/KC274 T1 (A3727) | 21.0 ±0.12 | 16.0 ± 0.53 | 0.44 ± 0.04 | Fig 4d |
| CCR2p-V-LUC F1 | CCR2p:AC  T1 (A3433) | UAS:JN/KC274 T1 (A3739) | 22.0 ± 0.35 | 10.3 ± 0.51* | 0.22 ± 0.01 | - |
| CCR2p-M-LUC F1 | CCR2p:AC  T1 (A3433) | UAS:JN/JR11-2 T1 (A3740) | 27.7 ± 0.71* | 4.8 ± 1.7* | 0.77 ± 0.21 | Fig 4d |
| CCR2p-M-LUC F1 | CCR2p:AC  T1 (A3433) | UAS:JN/JR11-2 T1 (A3729/37/40) | 20.5 ± 0.12* | 20.6 ± 0.81* | 0.42 ± 0.14 | - |
| DIN6p:LUC | n/a | n/a | 23.0 ± 0.29 | 7.5 ± 0.79 | 0.35 ± 0.12 | Fig 7a |
| DIN6p:LUC | n/a | n/a | 22.0 ± 0.24 | 11.3 ± 1.3 | 0.10 ± 0.04 | - |
| DIN6p-V-LUC F1 | DIN6p:AC  T2 (A3972) | UAS:JN/KC274 T1 (A3738) | 22.7 ± 0.16 | 7.5 ± 0.66 | 0.13 ± 0.05 | Fig 7a |
| DIN6p-V-LUC F1 | DIN6p:AC  T2 (A3972) | UAS:JN/KC274 T1 (A3727/38) | 22.1 ± 0.13 | 9.7 ± 1.0 | 0.20 ± 0.05 | - |
| SEN5p:LUC | n/a | n/a | 22.2 ± 0.24 | 9.9 ± 0.92 | 0.10 ± 0.02 | Fig 7b |
| SEN5p-V-LUC F1 (A4856-9) | SEN5p:AC/UAS:JN T3 (A3505) | KC274 | 22.6 ± 0.28 | 5.5 ± 2.6* | 0.21 ± 0.05 | Fig 7b |
| SEN5-M-LUC F1 (A4860) | SEN5p:AC/UAS:JN T3 (A3505) | JR11-2 | 22.5 ± 0.06 | 8.1 ± 0.42 | 0.15 ± 0.02 | Fig 7b |
| SEN5p-G-LUC F1 (A4851-55) | SEN5p:AC/UAS:JN T3 (A3505) | E1728 | 22.3 ± 0.35 | 8.6 ± 1.3 | 0.32 ± 0.06 | Fig 7b |

* Period and phase estimates significantly different from control by one-way ANOVA with Tukey’s post-hoc test (P < 0.05).

**Table S3.** Sequences of primers used in this study.

| **Primer Name** | **Primer Sequence (5’-3’)** | **Purpose** |
| --- | --- | --- |
| AC-XbaI_F | AAATCTAGAGGATCCCCGGTACCATG | GWp:AC construct |
| AC-SpeI_R | AAACTAGTTTACACGGCGATCTTTCCG | GWp:AC construct |
| PRR7p_F | CACCTGTCGATATGTCCGAGTGGT | PRR7:AC construct |
| PRR7p_R | CACACCAACTCTGCTTCGCTGAA | PRR7:AC construct |
| CCR2p-HindIII_F | AAAAAAGCTTAGCGAGCCATCTTCATAC | CCR2:AC construct |
| CCR2p-HindIII_R | AAAAAAGCTTTGAAATTTGAAAAGAAGATCTA | CCR2:AC construct |
| DIN6p_F | CACCCTTAACTATAACACAAAGAC | DIN6:AC construct |
| DIN6p_R | GTTTTTTTTTTGAAGAAAGTGAA | DIN6:AC construct |
| SEN5p_F | CACCACCGTGTCTCTCAAGTTA | SEN5:AC construct |
| SEN5p_R | CGTTTTCTTTGTCTTTGATTAATCC | SEN5:AC construct |
| JN-BamHI_F | AAAGGATCCCCAAAGGTACCATGTAC | UAS:JN construct |
| JN-SacI_R | AAAGAGCTCTTATCCATCCTTGTCAATCAA | UAS:JN construct |
| LUC+-XbaI_F | AAATCTAGAATGGAAGACGCCAAAAACAT | pEarleyGate301-LUC+ construct |
| LUC+-XbaI_R | ATATCTAGATTACACGGCGATCTTTCCG | pEarleyGate301-LUC+ construct |
| AD1 | (AGCT)GTCGA(GC)(AT)GA(AGCT)A(AT)GAA | TAIL PCR |
| GAL4131REV | GACACTTGGCGCACTTCGGCTTCTTC | TAIL PCR |
| GAL499REV | CACTTGAGTTCTTGAGGCGGGCAGAT | TAIL PCR |
| GAL445REV | AGCTTCATTGTTGGATCCGGTTCTCT | TAIL PCR |
| LB5102FWD | GGAACAACACTCAACCCTATCTCGGG | TAIL PCR |
| LB5147FWD | GGGATTTTGCCGATTTCGGAACCACC | TAIL PCR |
| LB5199FWD | GCAAACCAGCGTGGACCGCTTGCTGC | TAIL PCR |
| At5g65590_F | TCACCGTTGTCACTCTCGAA | sequence T-DNA flanking region |
| At1g75710_F | GTCACATGATCGTTGACCCG | sequence T-DNA flanking region |
| At3g51910_R | TGCAACCGTTACCAAAACCC | sequence T-DNA flanking region |
| At5g15980_F | GGACTGAACGAGGAGGAACA | sequence T-DNA flanking region |
| CCA1_F | GATGATGTTGAGGCGGATG | qRT-PCR |
| CCA1_R | TGGTGTTAACTGAGCTGTGAAG | qRT-PCR |
| TOC1_F | TCTTCGCAGAATCCCTGTGAT | qRT-PCR |
| TOC1_R | GCTGCACCTAGCTTCAAGCA | qRT-PCR |
| GAL4_F | GACATCTGCCGCCTCAAG | qRT-PCR |
| GAL4_R | GCGACACTCCCAGTTGTTCT | qRT-PCR |
| IPP2_F | GTATGAGTTGCTTCTCCAGCAAAG | qRT-PCR |
| IPP2_R | GAGGATGGCTGCAACAAGTGT | qRT-PCR |
| PP2AA3_F | TAACGTGGCCAAAATGATGC | qRT-PCR |
| PP2AA3_R | GTTCTCCACAACCGCTTGGT | qRT-PCR |
| UAS:nLUC_F | GAAGAGATACGCCCTGGTTCCT | qRT-PCR |
| UAS:nLUC_R | CCGATAAATAACGCGCCCAA | qRT-PCR |
| TOC1p:cLUC_F | TTGCTCCAACACCCCAACAT | qRT-PCR |
| TOC1p:cLUC_R | TCATCGTCTTTCCGTGCTCC | qRT-PCR |
| GAL4FP | GACATCTGCCGCCTCAAG | *in situ* probe synthesis |
| GAL4RP | GCTCGAGACGGTCAACTG | *in situ* probe synthesis |
| M13 (-40) | GTTTTCCCAGTCACGAC | *in situ* probe synthesis |
