# Supplementary material for: Combining GAL4 GFP enhancer trap with split luciferase to measure spatiotemporal promoter activity in Arabidopsis
Source: Plant J. 2019 Dec 3;102(1):187–98. doi: 10.1111/tpj.14603 (PMC7217008; doi:10.1111/tpj.14603)
Supplement: Supplementary file 2 [file TPJ-102-187-s002.docx]

**Table S1.** List of published GAL4 enhancer trap lines compatible with the ETSLA system.

**Table S2.** Summary of transgenic lines used in this study.

**Table S3.** Sequences of primers used in this study.
